# The Notification of Puerperal Fever

**Published:** 1913-07-05

**Authors:** 


### THE NOTIFICATION OF PUERPERAL FEVER.

### Some Arguments in Favour of its Abolition.

From time to time the provisions of the Public Health Acts as to notification of infective diseases have been extended by administrative authorities to cover maladies other than those to which compulsory notification was originally applicable by legislation. Some of these are diseases the infective nature of which has only been proved within comparatively recent years, such as epidemic poliomyelitis, cerebro-spinal fever, and ophthalmia neonatorum. Others, for example tuberculosis, though long recognised as of bacterial origin, were excluded from the original list of notifiable diseases for reasons of expediency, such as the then backward state of public opinion concerning them.

With the general policy of extending notification to every infective, and especially every epidemic, disease all modern expert opinion agrees. Thus measles is not yet notifiable in London, though public health authorities are practically unanimous in advocating that it should be; and it is probably only a matter of time before this desirable reform is instituted.

There is, however, one notifiable disease which might with advantage be deleted from the schedule, and that is "puerperal fever," so called. Only a couple of generations ago this condition was regarded as an epidemic disease of mysterious origin—or rather of disputed origin—and compulsory notification was quite justifiable in the light of what was then known about it. Nowadays it is universally admitted that puerperal fever, as a single definite disease, has no existence at all. Any sort of septic infection in any part of the genital tract may give rise to symptoms and signs of fever in a woman who has lately been through the ordeal of child-birth; and the treatment of this complication depends entirely on the situation of the infection and its character. Strictly speaking, any rise of temperature in a lying-in woman constitutes puerperal fever; but, of course, no one would apply this term to a temporary small rise above normal due, let us say, to constipation. The practical point is, however, that puerperal fever is not a definite disease, such as scarlet fever or typhoid fever; but merely a symptom of many different conditions.

Furthermore, a considerable section of the intelligent public is now aware that rigid antisepsis in the conduct of labour is an almost absolute precaution against puerperal fever; indeed, many of the public now believe that puerperal fever must necessarily be the result of negligence on the part of the obstetrician. It may be granted that puerperal sepsis diminishes in proportion as the strict observance of asepsis and antisepsis amongst obstetricians increases; but even the most expert and conscientious Listerian cannot guarantee complete immunity from puerperal fever in his patients. Knowing, however, that in the public mind some degree of odium and loss of reputation attaches to anybody in whose practice a case of puerperal fever occurs, doctors are extremely unwilling to admit that this condition exists and to follow out the law of the land by notifying it. The consequence is that the statistics dealing with the incidence of puerperal fever are misleading to a degree. As a rule only those cases get notified where the possibility of a fatal ending is by no means remote, or where admission to some public hospital is secured for the patient. The result is that the death-rate, as it appears from public health records, is somewhere in the neighbourhood of 40 to 50 per cent. of the cases; whereas if all cases were included it would probably not exceed 5 to 10 per cent., and might even be much less than that.

The present writer can call to mind a good many cases at least which were never notified as they should have been on a rigid interpretation of the law. Doubtless most practitioners could parallel these cases. A brief statement concerning four of them may be of use.

Some few years ago the writer admitted to hospital a woman desperately ill with acute septicæmia, two or three days only after her delivery. Despite all treatment she died in a few hours. He then learnt that another woman, delivered by the same practitioner, had died less than a week before with very similar symptoms; a death certificate was given to the relatives by this attending practitioner, stating that the cause of death was pneumonia. There can be little doubt that this also was a case of acute puerperal septicæmia, and it went not merely unnotified, but also entirely unregistered. The next case was one which occurred in the private practice of a London consulting obstetrician of good reputation. Death was officially ascribed to typhoid fever, but it was admitted subsequently that the real cause was puerperal sepsis.

The other two cases illustrate an opposite difficulty—that of deciding whether a border-line case should or should not be regarded as "puerperal fever." The writer a few months ago encountered a case of mild cystitis during the puerperium, lasting about a week. The temperature ranged between normal and two degrees over normal, and there was absolutely no cause for it other than the cystitis: the symptoms yielded to simple drug treatment without any necessity for local measures. The case was not notified; and probably no one would contend that it ought to have been. Yet in absolute strictness it was a case of "puerperal fever," because there is no hard-and-fast line which defines what is and what is not a degree of fever which should be notifiable. Then, again, he lately saw with a colleague a case in which, after a fortnight of normal convalescence, a lying-in woman developed a temperature five degrees over normal, together with other somewhat alarming symptoms of sapræmic absorption. An intra-uterine douche brought away a small clot, the temperature fell rapidly to normal, and subsequent convalescence was uninterrupted. Here, again, the practitioner did not notify the case, though it was certainly one of "puerperal fever," nor does it appear that the advantage of doing so, from a public health standpoint, would have outweighed the obvious disadvantages attaching to such a course.

What then should be done? In the opinion of the writer, and of much higher authorities than he, puerperal fever should be struck off the list of notifiable diseases altogether. The arguments in favour of this course have been very ably marshalled by Dr. V. Bonney.* He points out that the object of notification is to facilitate the segregation of cases of disease infectious to the community at large; to enable the sanitary authorities to take steps to disinfect the dwellings of such patients; and to permit of better investigation into the causes underlying the occurrence of epidemics. In all of these particulars notification fails in the case of puerperal sepsis; its only effect is to cast an often unmerited stigma on the obstetrician, and to create a consequent disinclination to comply with the law, or even to recognise the disease unless it becomes extreme.

* *A System of Treatment.* Edited by Latham and English. Vol. iv., p. 322.

In view of the bacteriology and pathology of child-bed fever, there is much less reason for its notification than in the case of wound sepsis after surgical operations, an occurrence far more within the control of the medical man.

Notification is, in fact, as the same author says, a remnant from the days when the disease was held to be due to some mysterious contagion, the method of whose spread was unexplained and unpreventable. He suggests that compliance with this out-of-date regulation is only reasonable and necessary when by reason of the bacteriological examination and the clinical picture it is apparent that the patient is suffering from infection by a virulent organism. The conceivable objection to its abolition is that one great incentive to obstetric carefulness and cleanliness might be abolished with it. But it has been shown, for one thing, that the vast majority of the cases, severe and trivial alike, are never notified as matters stand; and for another, we may remark that the worry and anxiety of having a case of puerperal sepsis on one's hands are a far greater incentive to perfect Listerian technique than the comparatively minor detail of complying with the public health laws as regards notification.

